# Bifid triple viable preparation combined with enteral nutrition as a supportive treatment for acute ischemic stroke: a systematic review and meta-analysis

**DOI:** 10.3389/fmicb.2024.1408960

**Published:** 2024-07-10

**Authors:** Yumeng Kong, Yunfeng Yu, Juan Deng, Rong Yu, Xiu Liu

**Affiliations:** ^1^School of Pharmacy, Hunan University of Chinese Medicine, Changsha, Hunan, China; ^2^School of Traditional Chinese Medicine, Hunan University of Chinese Medicine, Changsha, Hunan, China; ^3^The Third Hospital of Changsha, Changsha, Hunan, China

**Keywords:** probiotics, bifid triple viable preparation, supportive treatment, acute ischemic stroke, meta-analysis

## Abstract

**Background:**

The benefits and risks of bifid triple viable preparations in patients with acute ischemic stroke (AIS) are still controversial. This study aimed to assess the efficacy and safety of bifid triple viable preparations in combination with enteral nutrition for the management of AIS.

**Methods:**

Eight public databases including China National Knowledge Infrastructure, China Biology Medicine, VIP, WanFang, EBSCO, PubMed, Cochrane Library, and Web of Science were searched for relevant clinical literature, published through January 2024. These data were then used in the present meta-analysis.

**Results:**

A total of 15 studies involving 1,544 patients were included in the meta-analysis. In terms of nutritional status, the results showed that compared with enteral nutrition alone, the bifid triple viable preparation combination group increased the levels of total protein (mean difference [MD], 5.53; 95%confidence interval [CI], 1.94–9.12; *p* = 0.003), albumin (MD, 4.01; 95%CI, 2.96–5.06; *p* < 0.00001), prealbumin (MD, 23.08; 95%CI, 16.22–29.95; *p* < 0.00001), hemoglobin (MD, 9.31; 95%CI, 6.34–12.27; *p* < 0.00001), and transferrin (MD, 0.64; 95%CI, 0.23–1.05; *p* = 0.002); in terms of neurological function, it improved the Glasgow Coma Scale (MD, 2.09; 95%CI, 0.69–3.49; *p* = 0.003), National Institute of Health Stroke Scale (MD, −3.07; 95%CI, −3.73 to −2.40; *p* < 0.00001), and Neurological Disability Score (MD, −6.68; 95%CI, -7.29 to −6.08; *p* < 0.00001); in terms of intestinal barrier function, it reduced the levels of endotoxin (MD, −0.55; 95%CI, −0.71 to −0.39; *p* < 0.00001), D-lactic acid (MD, −3.17; 95%CI, −4.07 to −2.26; *p* < 0.00001), diamine oxidase (MD, −4.39; 95%CI, −6.20 to −2.57; *p* < 0.00001), and endothelin (MD, −21.35; 95%CI, −27.86 to −14.83; *p* < 0.00001); in terms of immune function, it increased the levels of immunoglobulin G (MD, 1.01; 95%CI, 0.20–1.82; *p* = 0.01) and immunoglobulin M (MD, 0.16; 95%CI, 0.02–0.30; *p* = 0.03). Additionally, it reduced the incidence of pulmonary infection, vomiting, constipation, and diarrhea, while there were no significant differences in total adverse events, abdominal distension, anorexia, reflux, gastrointestinal bleeding, or electrolyte disturbance.

**Conclusion:**

The addition of bifid triple viable preparation to enteral nutrition improved the nutritional status, neurological function, intestinal barrier function, and immune function of patients with AIS, and reduced the risk of infection and gastrointestinal events.

## 1 Introduction

Ischemic stroke (IS), a disease caused by various factors, leads to disorders of the cerebral circulatory system, subsequently resulting in localized ischemic necrosis or softening of the brain tissue (Liu and Xie, 2014). IS has high incidence, disability, mortality, and recurrence rates, and is the second leading cause of death and the third leading cause of disability worldwide (Tang et al., 2023). IS is reported to account for more than 70% of all stroke cases, and has become a major global public health problem (Wu et al., 2019). Due to a narrow treatment window, limited applicability of treatment methods, poor treatment outcomes, and drug-related adverse events, all of which make treatment difficult for clinicians to navigate (Yang et al., 2021). A series of complex neuropathological events, including inflammation, ion imbalance, cell apoptosis, amyloid production, excitotoxicity, and oxidative stress are involved in the pathogenesis of IS (Pluta et al., 2021), posing significant challenges to the management of IS, especially acute ischemic stroke (AIS). Moreover, the stress response in AIS patients can lead to severe malnutrition, heightened complications, and a worsened prognosis (Bouziana and Tziomalos, 2011). This malnutrition further amplifies stress reactions, consequently escalating the likelihood of complications (Zielińska-Nowak et al., 2021). As research has progressed, the microbiota-gut-brain axis (MGBA), a bidirectional communication pathway between the gastrointestinal and central nervous systems, has garnered increasing attention, with researchers believing that gut microbiota may play an important role in the development or prevention of AIS by regulating the MGBA (Sinagra et al., 2021).

AIS disrupts the intestinal barrier, leading to dysbiosis of the gut microbiota and poor nutrient absorption, subsequently resulting in a poor prognosis in patients with AIS (Sabbouh and Torbey, 2018; Zhai et al., 2023). The gut microbiota and its metabolites also have an inverse effect on the occurrence, development, and outcome of AIS, thereby forming a vicious cycle of mutual pathological interference (Li Z. et al., 2021). Probiotics have the potential to improve the prognosis of AIS by regulating the gut microbiota, owing to the role they play in restoring intestinal barrier function, slowing the deterioration of nutritional status, and enhancing cellular immunity (Zeng, 2015). Probiotics are microorganisms that provide significant health benefits when ingested in adequate amounts (Hill et al., 2014), and have long been used for the treatment and prevention of various diseases (Wiegers et al., 2023). Common probiotic preparations include bifid triple and quadruple viable preparations as well as *Bacillus subtilis* bivalent combination preparations. Previous meta-analyses have shown that probiotics improve enteral nutrition and immune function, and reduce the risk of adverse gastrointestinal events in stroke patients (Liu et al., 2021; Zhong et al., 2021; Chen et al., 2022). These meta-analyses, however, focused only on the overall effects of probiotics on stroke, and did not adequately explain the individual effects of probiotics on patients with AIS. Additionally, they did not independently analyze the role of different strains of probiotics in stroke; therefore, it is necessary to conduct new meta-analyses to evaluate the value of specific probiotic formulations in AIS.

The Bifid triple viable preparation is available in two dosage forms: tablet and capsule. The main components of the capsule are *Bifidobacterium longum*, *Lactobacillus acidophilus*, and *Enterococcus faecalis*, while the main components of the tablet are *Bifidobacterium longum*, *Lactobacillus bulgaricus*, and *Streptococcus thermophilus*. Among them, microorganisms from Lactobacillus and Bifidobacterium genera constitute the most frequently used human probiotics (Brodmann et al., 2017; Nogacka et al., 2019). The combination of Lactobacillus and Bifidobacterium is capable of long-term maintenance and improvement in regulating the human intestinal microbiota (Zhang, 2022). Therefore, we focus on the impact of bifid triple viable preparation containing Lactobacillus and Bifidobacterium on the prognosis of AIS. In the present study, we performed a meta-analysis to quantitatively assess the efficacy and safety of a bifid triple viable preparation combined with enteral nutrition for the management of AIS, with the aim of providing new ideas for the clinical management of AIS.

## 2 Materials and methods

The protocol for the present study followed the Preferred Reporting Items for Systematic Reviews and Meta Analyses (PRISMA) guidelines (Radua, 2021), and was registered in the PROSPERO (registration number: CRD42024552162).

### 2.1 Literature search

We searched the China National Knowledge Infrastructure, China Biology Medicine, VIP, WanFang, PubMed, EBSCO, Cochrane Library, and Web of Science databases for relevant clinical trials published before January 2024. Taking the PubMed database as an example, the search field was [Title/Abstract], and the search formula was as follows: (Probiotic OR Bifidobacterium OR Bifidobacteria OR Bacillus bifida OR Yeast OR *Saccharomyces cerevisiae* OR Saccharomyces italicus OR Saccharomyces oviformis OR S cerevisiae OR *S. cerevisiae* OR Saccharomyces uvarum var. melibiosus OR *Candida robusta* OR Saccharomyces capensis OR *Lactobacillus acidophilus* OR *Lactobacillus amylovorus* OR Lactobacill* OR lactic acid bacteria OR *Clostridium butyricum* OR Slaysophilus OR Bacillus OR Natto Bacteria OR Streptococcus thermophiles OR Enterococcus) and (Ischemic Stroke OR Ischemic Strokes OR Ischaemic Stroke OR Ischaemic Strokes OR Acute Ischemic Stroke OR Acute Ischemic Strokes OR AIS OR Brain Ischemia OR Middle Cerebral Artery occlusion OR MCA OR Large Vessel Occlusion OR LVO OR Brain Infarction OR Cerebral Infarction). The search terms included components of bifid triple viable and other probiotic strains to ensure the comprehensiveness of the literature search.

### 2.2 Inclusion and exclusion criteria

The inclusion criteria followed the PICOS principle. (1) Participants: patients diagnosed with AIS by cranial magnetic resonance imaging (Chinese Society of Neurology, Chinese Stroke Society, 2018). (2) Intervention: the experimental group received enteral nutrition and bifid triple viable preparation. (3) Comparison: the control group received enteral nutrition only. (4) Outcomes: the primary outcome measures were nutritional status (total protein [TP], albumin [ALB], prealbumin [PA], hemoglobin [Hb], transferrin [TRF]), and the secondary outcome measures included neurological function (National Institute of Health Stroke Scale [NIHSS], Glasgow Coma Scale [GCS], Neurological Disability Score [NDS]), intestinal barrier function (endotoxin, D-lactic acid, diamine oxidase [DAO], endothelin [ET]), and immune function (immunoglobulin G, immunoglobulin M, and immunoglobulin A [IgG, IgM, IgA]). The safety outcome measures included all adverse events mentioned in the literature that could be meta-analyzed (total adverse events, pulmonary infection, abdominal distension, anorexia, vomiting, reflux, constipation, diarrhea, gastrointestinal bleeding, electrolyte disturbance). (5) Study design: randomized controlled trial.

The exclusion criteria were as follows: (1) literature for which the data was not available would be excluded; (2) duplicate publications would be screened out, retaining only the most comprehensive and recent data; (3) literature not meeting the diagnostic criteria for AIS as defined by cranial magnetic resonance imaging would be excluded; (4) studies failing to meet rigorous quality assessment standards or with significant research design flaws would be excluded; and (5) trials with unclear intervention details or deviations from the specified plans would be excluded.

### 2.3 Literature screening

Two researchers, Kong YM and Deng J, independently screened the literature according to the established inclusion and exclusion criteria using NoteExpress version 3.0 (Aiqinhaiyuezhi Technology Co., Beijing). The duplicates were sequentially excluded, as were literature not related to the study topic and literature lacking complete data, after which the included literature were finalized.

### 2.4 Data extraction

The aforementioned researchers compiled the basic information for each study, including the following: author name(s); country; sources of participants; sample size; male ratio; average age; average ALB; intervention; and treatment duration. These information were then input into a basic information table created by Excel 2010.

### 2.5 Risk of bias assessment

Kong YM and Deng J assessed the risk of bias for each study using the Cochrane tool. Assessment items included random generation, allocation concealment, blinding, incomplete outcome data, selective reporting, and other biases. The evaluation ratings were categorized as low, unclear, or high risk.

### 2.6 Statistical analysis

First, we conducted a meta-analysis using the RevMan 5.3. Mean difference (MD) and 95% confidence interval (CI) were used as the effect sizes for continuous variables, whereas risk ratio (RR) and 95% CI were used as the effect sizes for dichotomous variables. Employing a random effects model for all meta-analyses, irrespective of the presence of low heterogeneity, represents a robust and cautious approach. This method allows for the consideration of potential differences between studies, accounting for both within-study and between-study variations, resulting in more generalizable and conservative estimates of the effect sizes. For the effect models, a *p*-value <0.05 was considered statistically significant. Second, we conducted a leave-one-out sensitivity analysis to assess the robustness of the results. After sequentially excluding each data point and reanalyzing it, the results were considered robust if the combined effect size did not significantly change. Third, we performed a subgroup analysis to evaluate the impact of different doses and treatment duration on ALB. Fourth, we conducted Egger’s test using Stata 15.0 (StataCorp LLC, United States). A *p*-value >0.1 in Egger’s test was considered indicative of no publication bias.

## 3 Results

### 3.1 Results of literature screening

**Figure 1:**
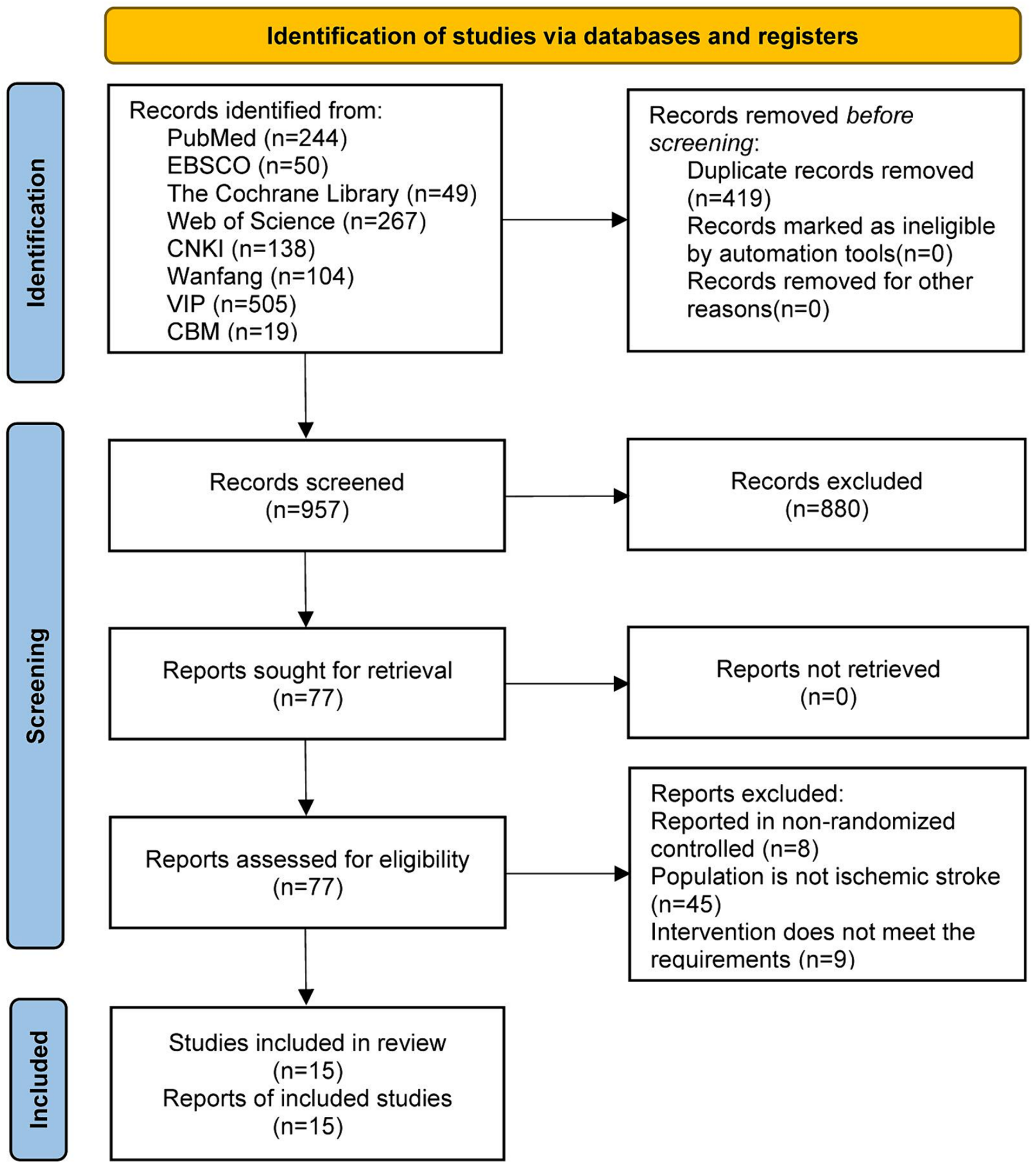
Literature screening process diagram.

A total of 1,376 articles were retrieved from the initial searches. We excluded 419 duplicate articles and 942 unrelated articles that did not meet our inclusion criteria. In total, 15 articles published between 2016 and 2023 were included in the present meta-analysis (Pang, 2016; Zhao, 2017; Yang et al., 2018; Chen et al., 2019; Huang and Jiang, 2019; Yu, 2020; Gao, 2021; Li L. et al., 2021; Wan et al., 2021a,b; Wang et al., 2021; Wang, 2021; Yang, 2021; Li et al., 2023; Zhang et al., 2023). A flow diagram of the study is shown in Figure 1.

### 3.2 Basic characteristics of included studies

A total of 15 clinical trials involving 1,544 Chinese patients were included in the present meta-analysis. Among them, 771 received enteral nutrition and 773 received a bifid triple viable preparation combined with enteral nutrition. All of the studies were from China and the participants were Chinese. The mean male ratio of the participants was 57.6%, the mean age was 66.9 years, and the mean serum ALB level was 33.9 g/L. Additionally, bifid triple viable preparation was administered at doses ranging from 0.84 g/d to 6.00 g/d, with a duration of treatment ranging from 2 weeks to 4 weeks. The basic characteristics of the included studies are presented in Table 1.

**Table 1:** Basic characteristics of the included studies.

Author name	Country	Sources of participants	Stroke Stage	Sample size (E/C)	Male (%)	Age (years)	ALB (g/L)	Probiotics	Treatment duration (weeks)
Chen JY 2019	China	Chinese	AIS	35/34	60.9	71.0	/	Bifid triple viable preparation NGT 0.42 g tid	2
Gao J 2021	China	Chinese	AIS	24/24	60.4	59.2	/	Bifid triple viable preparation PO 2.00 g tid	2
Huang J 2019	China	Chinese	AIS	42/40	52.4	70.1	39.6	Bifid triple viable preparation NGT 0.84 g bid	3
Li L 2021	China	Chinese	AIS	45/45	48.9	68.6	31.7	Bifid triple viable preparation NGT 0.63 g tid	4
Li Y 2023	China	Chinese	AIS	210/210	66.4	65.2	/	Bifid triple viable preparation NGT 0.42 g tid	2
Pang XE 2016	China	Chinese	AIS	48/48	54.2	71.9	37.2	Bifid triple viable preparation NGT 0.63 g tid	4
Wan L 2021–1	China	Chinese	AIS	42/41	60.2	57.5	28.1	Bifid triple viable preparation NGT 0.63 g tid	2
Wan L 2021–2	China	Chinese	AIS	28/30	/	/	38.5	Bifid triple viable preparation NGT 2.00 g tid	2
Wang J 2021	China	Chinese	AIS	51/51	52.9	67.9	37.2	Bifid triple viable preparation NGT 0.42 g tid	2
Wang N 2021	China	Chinese	AIS	48/48	55.2	68.9	37.2	Bifid triple viable preparation NGT 0.42 g tid	2
Yang GH 2021	China	Chinese	AIS	40/40	55.0	73.2	27.5	Bifid triple viable preparation NGT 0.42 g bid	4
Yang JY 2018	China	Chinese	AIS	30/30	32.0	65.8	38.0	Bifid triple viable preparation NGT 0.63 g tid	4
Yu WL 2020	China	Chinese	AIS	53/53	55.7	71.6	27.3	Bifid triple viable preparation NGT 0.42 g bid	4
Zhang TT 2023	China	Chinese	AIS	40/40	56.3	56.9	38.6	Bifid triple viable preparation NGT 2.00 g tid	1
Zhao J 2017	China	Chinese	AIS	37/37	58.1	70.0	27.4	Bifid triple viable preparation NGT 0.63 g tid	4

AIS, acute ischemic stroke; ALB, albumin; E, experimental group; C, control group.

### 3.3 Bias risk assessment

**Figure 2:**
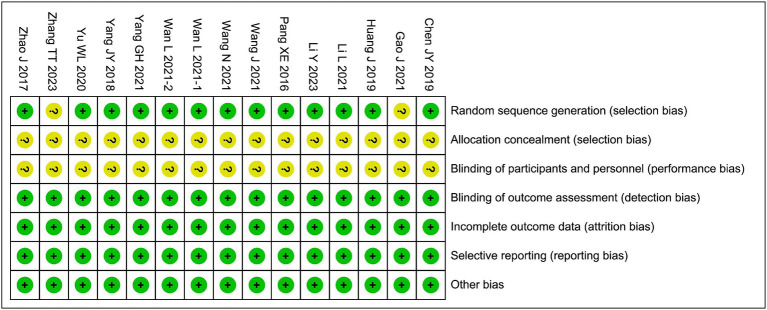
Risk of bias graph.

Among the included studies, the randomization methods of 2 studies were assessed as having an unclear risk of bias, and the allocation concealment and blinding of participants and personnel in all 15 studies were assessed as having an unclear risk of bias. The risks of bias in the remaining areas were considered low, as shown in Figure 2.

### 3.4 Meta-analysis results

#### 3.4.1 Nutritional status

**Figure 3:**
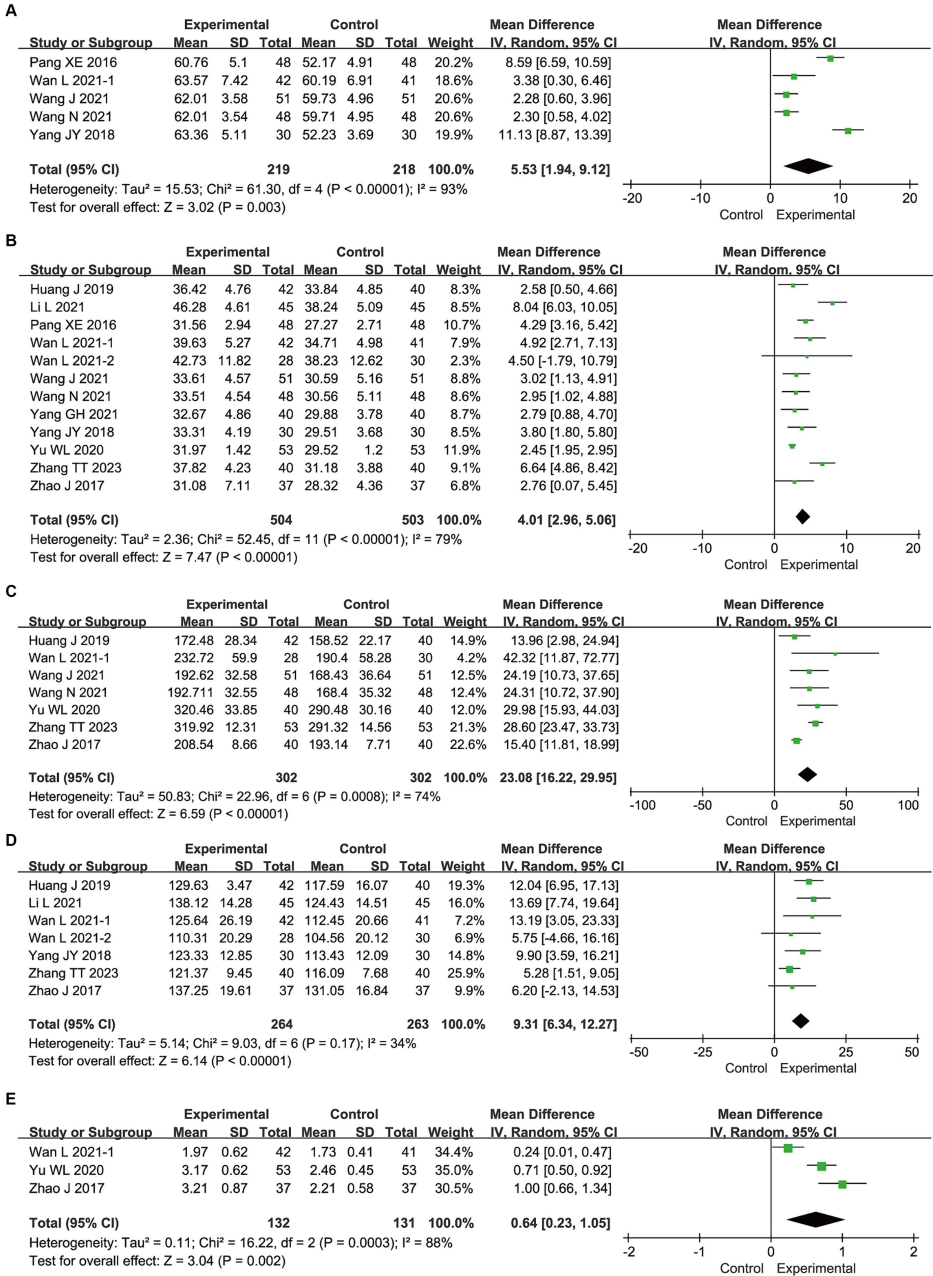
Meta-analysis results for the nutritional status of bifid triple viable preparation in acute ischemic stroke. **(A)** Total protein; **(B)** Albumin; **(C)** Prealbumin; **(D)** Hemoglobin; **(E)** Transferrin.

Figure 3 presents the meta-analysis results for nutritional status. Compared with enteral nutrition alone, the combination of bifid triple viable preparation and enteral nutrition significantly increased the levels of TP (MD, 5.53; 95% CI, 1.94–9.12; *p* = 0.003), ALB (MD, 4.01; 95% CI, 2.96–5.06; *p* < 0.00001), PA (MD, 23.08; 95% CI, 16.22–29.95; *p* < 0.00001), Hb (MD, 9.31; 95%CI, 6.34–12.27; *p* < 0.00001), and TRF (MD, 0.64; 95% CI, 0.23–1.05; *p* = 0.002) in patients with AIS. Sensitivity analysis showed that the results for TRF were not robust, whereas the remaining results were robust. After excluding the study by Yu (2020), TRF no longer showed significant differences (MD, 0.61; 95% CI, −0.14–1.35; *p* = 0.11).

#### 3.4.2 Neurological function

Supplementary Figure S1 presents the results of the meta-analysis of neurological function. Compared with enteral nutrition alone, the combination of bifid triple viable preparation and enteral nutrition significantly reduced the NIHSS (MD, −3.07; 95% CI, −3.73 to −2.40; *p* < 0.00001) and NDS (MD, −6.68; 95% CI, −7.29 to −6.08; *p* < 0.00001) and increased the GCS (MD, 2.09; 95% CI, 0.69–3.49; *p* = 0.003). Sensitivity analysis showed that all of these outcomes were robust.

#### 3.4.3 Intestinal barrier function

Supplementary Figure S2 presents the results of the meta-analysis of intestinal barrier function. Compared with enteral nutrition alone, the combination of bifid triple viable preparation and enteral nutrition significantly reduced the levels of endotoxin (MD, −0.55; 95% CI, −0.71 to −0.39; *p* < 0.00001), D-lactic acid (MD, −3.17; 95% CI, −4.07 to −2.26; *p* < 0.00001), diamine oxidase (MD, −4.39; 95% CI, −6.20 to −2.57; *p* < 0.00001), and endothelin (MD, −21.35; 95%CI, −27.86 to −14.83; *p* < 0.00001) in patients with AIS. Sensitivity analysis showed that all of these outcomes were robust.

#### 3.4.4 Immune function

Supplementary Figure S3 presents the meta-analysis results of immune function. Compared with enteral nutrition alone, the combination of bifid triple viable preparation and enteral nutrition significantly increased the levels of IgG (MD, 1.01; 95% CI, 0.20–1.82; *p* = 0.01) and IgM (MD, 0.16; 95% CI, 0.02–0.30; *p* = 0.03) in patients with AIS, while the IgA level was comparable (MD, 0.05; 95% CI, –0.01–0.10; *p* = 0.12). Sensitivity analysis showed that all of these outcomes were not robust. After excluding the study by Gao (2021), IgG (MD, 1.43; 95% CI, –0.38–3.24; *p* = 0.12) and IgM (MD, 0.39; 95% CI, –0.29–1.08; *p* = 0.26) no longer showed significant changes. After excluding the study by Wan et al. (2021b), however, IgA showed significant changes (MD, 0.03; 95% CI, 0.01–0.04; *p* = 0.0005).

#### 3.4.5 Safety endpoints

Table 2 shows the meta-analysis results of safety endpoints. Compared with enteral nutrition alone, the combination of bifid triple viable preparation and enteral nutrition significantly reduced pulmonary infection (RR, 0.52; 95% CI, 0.35–0.77; *p* = 0.001), vomiting (RR, 0.27; 95% CI, 0.10–0.70; *p* = 0.007), constipation (RR, 0.34; 95% CI, 0.16–0.74; *p* = 0.007), and diarrhea (RR, 0.27; 95% CI, 0.14–0.54; *p* = 0.0002) in patients with AIS, while the total adverse events (RR, 0.53; 95% CI, 0.27–1.06; *p* = 0.07), abdominal distension (RR, 0.62; 95% CI, 0.28–1.37; *p* = 0.24), anorexia (RR, 0.42; 95% CI, 0.14–1.28; *p* = 0.13), reflux (RR, 0.23; 95% CI, 0.05–1.08; *p* = 0.06), gastrointestinal bleeding (RR, 0.33; 95% CI, 0.03–3.07; *p* = 0.33), and electrolyte disturbance (RR, 0.27; 95% CI, 0.03–2.87; *p* = 0.28) ware comparable. The sensitivity analysis showed that the results for abdominal distension and constipation were not robust, whereas all of the remaining results were. After excluding the study by Zhao (2017), constipation no longer changed significantly (RR, 0.44; 95% CI, 0.17–1.11; *p* = 0.08). After excluding the study by Huang and Jiang (2019), abdominal distension did change significantly (RR, 0.51; 95% CI, 0.26–0.97; *p* = 0.04).

**Table 2:** Meta-analysis results for the safety endpoints of bifid triple viable preparation in acute ischemic stroke.

Outcome	Number of studies	Experimental (events/total)	Control (events/total)	I^2^/%	RR (95%CI)	*p* value
Total adverse events	3	32/139	58/139	60	0.53 (0.27, 1.06)	0.07
Pulmonary infection	5	18/209	42/209	0	0.52 (0.35, 0.77)	0.001
Abdominal distension	5	13/200	24/200	18	0.62 (0.28, 1.37)	0.24
Anorexia	3	4/121	10/123	0	0.42 (0.14, 1.28)	0.13
Vomiting	4	4/158	24/160	0	0.27 (0.10, 0.70)	0.007
Reflux	2	2/76	10/78	0	0.23 (0.05, 1.08)	0.06
Constipation	5	8/198	25/200	0	0.34 (0.16, 0.74)	0.007
Diarrhea	5	9/198	34/200	0	0.27 (0.14, 0.54)	0.0002
Gastrointestinal bleeding	2	0/87	2/85	0	0.33 (0.03, 3.07)	0.33
Electrolyte disturbance	2	2/99	10/99	53	0.27 (0.03, 2.87)	0.28

### 3.5 Subgroup analysis

The following subgroups were established based on the therapeutic dose: ≤ 1.0 g/d; 1.1–2.0 g/d; and > 2.0 g/d. The results showed that ≤1.0 g/d (MD, 2.47; 95% CI, 1.99–2.96; *p* < 0.00001), 1.1–2.0 g/d (MD, 4.08; 95% CI, 2.90–5.25; *p* < 0.00001), and > 2.0 g/d (MD, 6.48; 95% CI, 4.77–8.19; *p* < 0.00001) of bifid triple viable preparation significantly increased ALB levels in patients with AIS. The test for subgroup differences showed that *p* < 0.00001.

**Figure 4:**
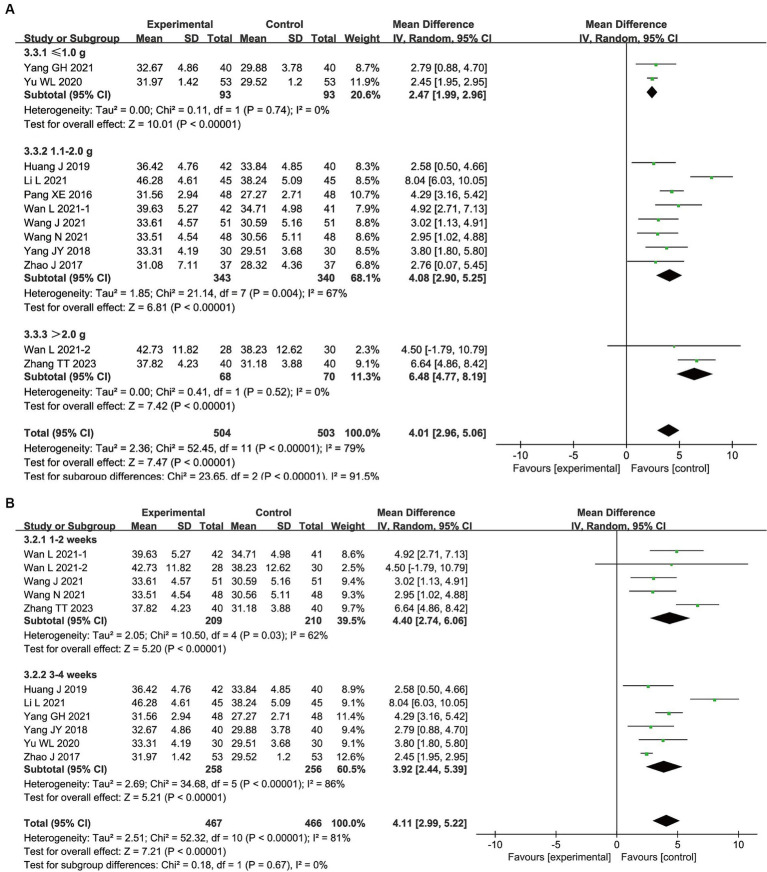
Subgroup analysis results for bifid triple viable preparation in acute ischemic stroke. **(A)** Therapeutic dose; **(B)** Duration of treatment.

Additionally, the subgroups of ‘1–2 weeks’ and ‘3–4 weeks’ were created based on the duration of treatment. The results showed that both 1–2 weeks (MD, 4.40; 95% CI, 2.74–6.06; *p* < 0.00001) and 3–4 weeks (MD, 3.92; 95% CI, 2.44–5.39; *p* < 0.00001) of bifid triple viable preparation significantly increased ALB levels in patients with AIS. The test for subgroup differences showed that *p* = 0.67. As shown in Figure 4.

### 3.6 Publication bias

**Figure 5:**
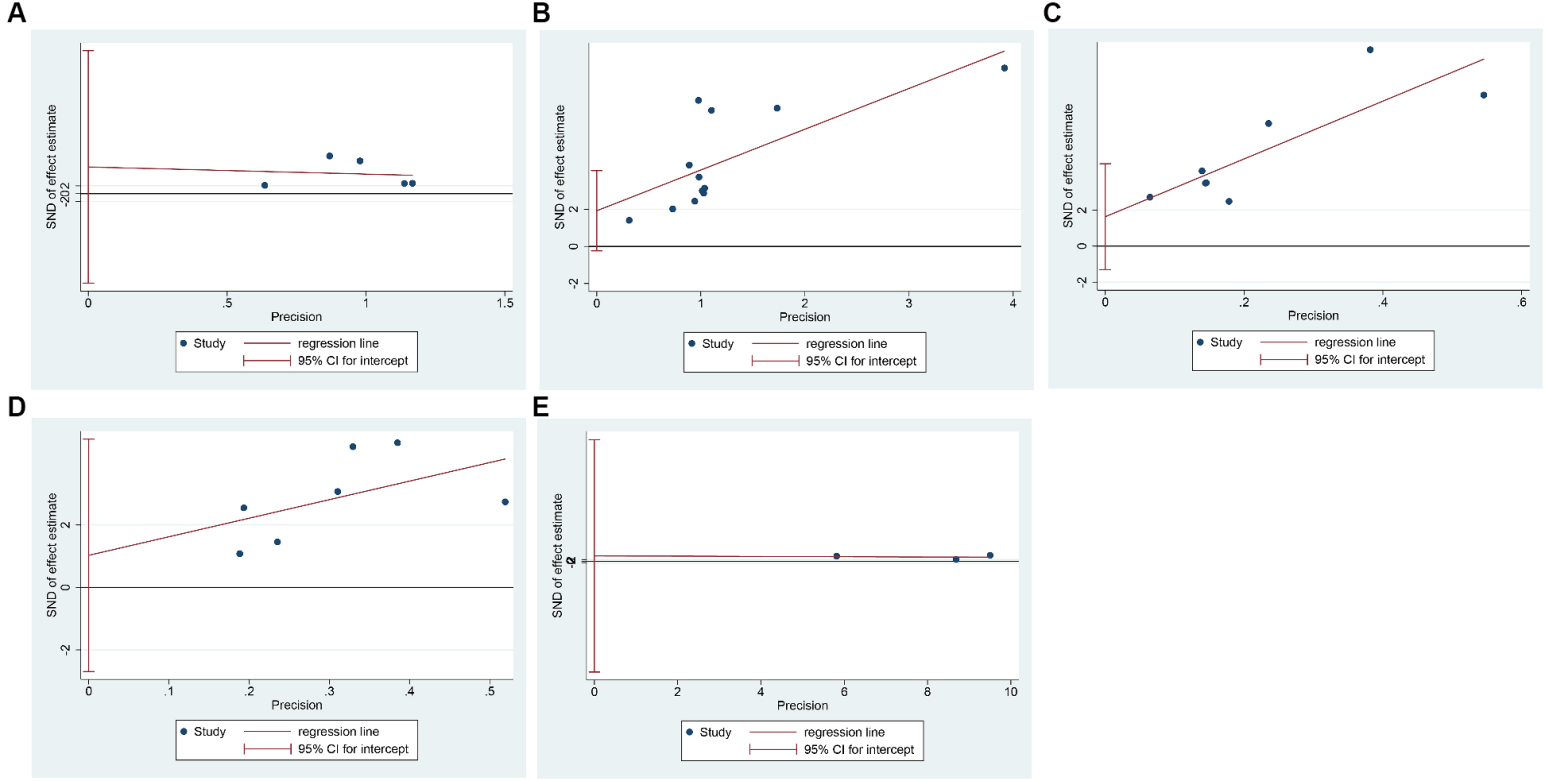
Egger’s test for publication bias. **(A)** Total protein; **(B)** Albumin; **(C)** Prealbumin; **(D)** Hemoglobin; **(E)** Transferrin.

We evaluated the publication bias of the primary outcome measures using Egger’s test. It showed that there was no publication bias in the results of TP (*p* = 0.517), PA (*p* = 0.222), Hb (*p* = 0.509), and TRF (*p* = 0.659), while there was publication bias in the results of ALB (*p* = 0.076), as shown in Figure 5.

## 4 Discussion

### 4.1 Efficacy analysis

In terms of nutritional status, the results of the present study showed that the combined use of bifid triple viable preparations increased the levels of TP, ALB, PA, Hb, and TRF in patients with AIS. The improvements in these indicators not only directly demonstrate the role of bifid triple viable preparation in improving nutritional status, but also indirectly reflect its potential to alleviate nerve damage. Three previous meta-analyses found that probiotics significantly increased the levels of TP, ALB, PA, and Hb in stroke patients (Liu et al., 2021; Zhong et al., 2021; Chen et al., 2022), supporting the findings of the present study. In contrast, the present meta-analysis also identified the benefits of the bifid triple viable preparation on the TRF levels, which further supported the value of the probiotics in improving the nutritional status of patients with AIS.

In terms of neurological function, the results of the present study indicated that the combined use of bifid triple viable preparation and enteral nutrition reduces the NIHSS and NDS scores and increases the GCS scores in patients with AIS. The results of a previous meta-analysis by Liu et al. (2021) are commensurate with our findings on GCS scores, while Chen et al. (2022) did not report the impact of probiotics on neurological function. Of note, the meta-analysis by Zhong et al. (2021) did not show any benefit to the use of bifid triple viable preparation in terms of the NIHSS, which is in stark contrast with the results of the present study. This contradiction may be due to the different probiotic strains studied. Zhong et al. (2021) included various strains, such as *Bifidobacterium*, *Lactobacillus*, *Enterococcus*, *Streptococcus thermophilus*, and *Clostridium butyricum*, while the present study only evaluated bifid triple viable preparations. In the present study, we found that the bifid triple viable preparation provided additional benefits in regards to the NIHSS and NDS scores, indicating its potential for the stronger promotion of neurological repair.

In terms of intestinal barrier function, the results of the present study indicated that the combined use of bifid triple viable preparations and enteral nutrition reduced the levels of DAO, D-lactic acid, endotoxin, and endothelin in patients with AIS. The above indicators are commonly used to evaluate the intestinal barrier function, and the benefits observed reflect the advantages of the bifid triple viable preparation in repairing the intestinal barrier in patients with AIS. In previous meta-analyses, Chen et al. (2022) reported the benefits of probiotics on DAO and D-lactic acid for patients with IS, supporting the findings of the present study, while Liu et al. (2021) and Zhong et al. (2021) did not report endpoints related to the intestinal barrier. Additionally, the results of the present study showed that the use of bifid triple viable preparation significantly reduced the levels of endotoxin and endothelin, indicating its potential to alleviate endotoxin damage and improve intestinal barrier repair.

In terms of immune function, the results of the present study indicated that the combination of bifid triple viable preparations and enteral nutrition increased IgG and IgM levels in patients with AIS, with no significant impact on IgA levels. In previous meta-analyses, Chen et al. (2022) reported an increase in IgG and IgA levels in stroke patients treated with probiotics, whereas Liu et al. (2021) and Zhong et al. (2021) did not evaluate the effect of probiotics on immunoglobulins. Interestingly, the results of the present meta-analysis showed benefits for IgM with the use of bifid triple viable preparations, but without the IgA benefits reported by Chen et al. (2022). This difference may be due to variations in the bacterial strains and stroke types evaluated. Nevertheless, the results of both the present study and that of Chen et al. (2022) support the idea that probiotics significantly increase IgG levels, highlighting the role of the bifid triple viable preparation in the regulation of long-term immune function.

### 4.2 Safety analysis

The results of the present study showed that the total number of adverse events in the bifid triple viable preparation combination group were comparable to those in the enteral nutrition only group, suggesting that the bifid triple viable preparation has a favorable safety profile and does not increase the risk of additional adverse events. Specifically, pulmonary infection, vomiting, constipation, and diarrhea were significantly reduced in the bifid triple viable preparation combination group compared to the enteral nutrition only group, while abdominal distension, anorexia, reflux, gastrointestinal bleeding, and electrolyte disturbance were comparable. It means that bifid triple viable preparations can reduce the risk of pulmonary infection and some gastrointestinal adverse events in patients with AIS. Two previous meta-analyses reported benefits of probiotics in reducing the risk of pulmonary infection, constipation, diarrhea and vomiting (Zhong et al., 2021; Chen et al., 2022), and another meta-analysis reported benefits of probiotics in reducing gastrointestinal complication (Liu et al., 2021), which supports our findings. However, the study by Chen et al. (2022) also showed that probiotics reduced abdominal distension, while the study by Zhong et al. (2021) noted a reduction in reflux and gastrointestinal bleeding with probiotics, which is different from our results. These differences may be caused with the type of probiotic as well as the type and severity of stroke. Therefore, the effect of bifid triple viable preparation on abdominal distension, reflux and gastrointestinal bleeding needs to be further explored.

### 4.3 Dosage and duration analysis

The dosage subgroup analysis in the present study showed that bifid triple viable preparation at ≤1.0 g/day, 1.1–2.0 g/day, and > 2.0 g/day all significantly increased the ALB levels, suggesting that all doses of bifid triple viable preparation improved the prognoses of patients with AIS. Previous studies, however, have found that doses of *Bifidobacterium* at 10^9^ and 10^10^ CFU/day significantly reduced colonic tissue damage, whereas low doses did not achieve this benefit (Chen et al., 2020), which indicates that a sufficient dose is a key factor for *Bifidobacterium* and its combination preparations to achieve beneficial effects. The authors, therefore, recommend that the dosage of the bifid triple viable preparation for patients should be maintained at ≥1.0 g/day.

In terms of treatment duration, the administration of bifid triple viable preparations for 1–2 weeks and 3–4 weeks significantly increased ALB levels, suggesting that both short- and medium-term duration of bifid triple viable preparations achieved additional benefits. However, considering that the standard length of stay for the clinical course of IS is 21–28 days (Office of the National Health and Family Planning Commission, 2016), a treatment course of 3–4 weeks may be more in-line with clinical practice. Based on the current evidence, therefore, we recommend that patients with AIS take bifid triple viable preparation of ≥1.0 g/day and continue for at least 3–4 weeks.

### 4.4 Limitations and perspectives

Although the present study has enriched the clinical evidence for the use of bifid triple viable preparations in treating AIS, some limitations need to be acknowledged. First, the literature included in the present study did not report allocation concealment methods, which may pose a potential risk of selection bias. Additionally, the lack of reporting on the use of blinding increases the potential risk of implementation bias. Second, the included studies were all conducted in Chinese, with study subjects of Chinese descent, which reduces the generalizability of the results. Third, the average age of the participants included in the present meta-analyses ranged from 56.9–73.2 years, which does not fully reflect the situation of patients across all age groups. Fourth, although the results of the present study describe the benefits of bifid triple viable preparations in patients with AIS, the optimal dosage and duration of treatment remain unclear.

The authors recommend that future research make efforts to address these limitations: first, strictly control allocation concealment and blinding to provide higher quality evidence for the application of bifid triple viable preparations in patients with AIS patients; second, design multicenter clinical trial protocols to evaluate the effects of bifid triple viable preparations on patients with AIS with a variety of demographic characteristics; third, promote the progress of subgroup clinical studies to reveal the optimal dosage and duration of bifid triple viable preparations for the management of AIS.

## 5 Conclusion

The administration of bifid triple viable preparation in conjunction with enteral nutrition improved the nutritional status, neurological function, intestinal barrier function, and immune function of patients with AIS, and reduced infections and adverse gastrointestinal events, making it a potential adjunctive treatment strategy for AIS. The optimal timing, dosage, and duration of treatment, however, need to be explored in future studies.

## Data availability statement

The original contributions presented in the study are included in the article/Supplementary material, further inquiries can be directed to the corresponding authors.

## Author contributions

YK: Conceptualization, Data curation, Methodology, Supervision, Writing – original draft. YY: Data curation, Methodology, Writing – original draft. JD: Formal analysis, Writing – original draft. RY: Formal analysis, Writing – review & editing. XL: Conceptualization, Supervision, Writing – review & editing.
