# Supplementary material for: Bifid triple viable preparation combined with enteral nutrition as a supportive treatment for acute ischemic stroke: a systematic review and meta-analysis
Source: Front Microbiol. 2024 Jul 10;15:1408960. doi: 10.3389/fmicb.2024.1408960 (PMC11268325; doi:10.3389/fmicb.2024.1408960)
Supplement: Supplementary file 1 [file Table_1.DOCX]

Supplementary Material

Bifid triple viable preparation combined with enteral nutrition as a supportive treatment for acute ischemic stroke: a systematic review and meta-analysis

Yumeng Kong^1†^, Yunfeng Yu^2†^, Juan Deng^3^, Rong Yu^2*^, Xiu Liu^2*^


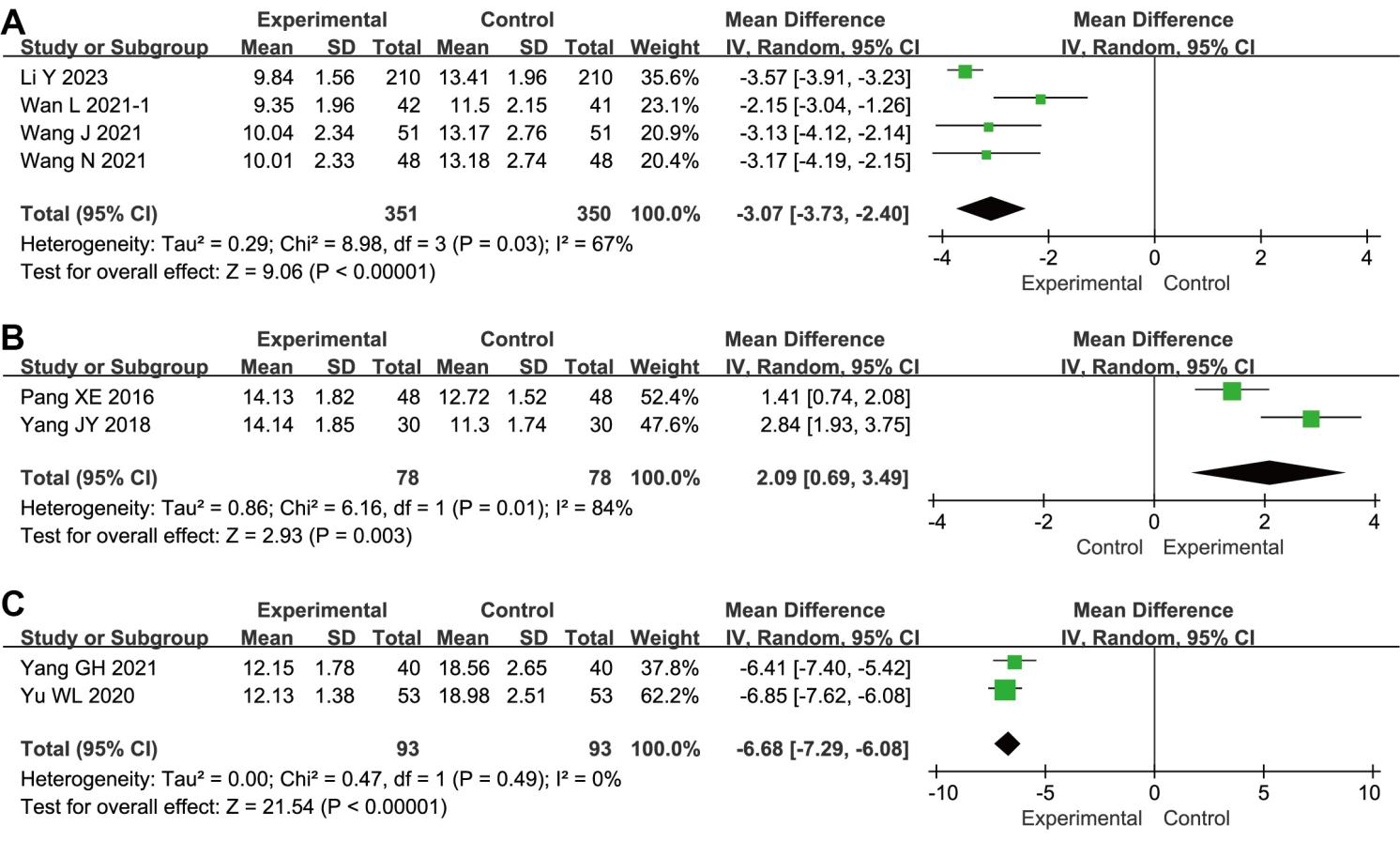


**Supplementary Figure S1.** Meta-analysis results for the neurological function of bifid triple viable preparation in acute ischemic stroke. (A) NIHSS; (B) GCS; (C) NDS.

**
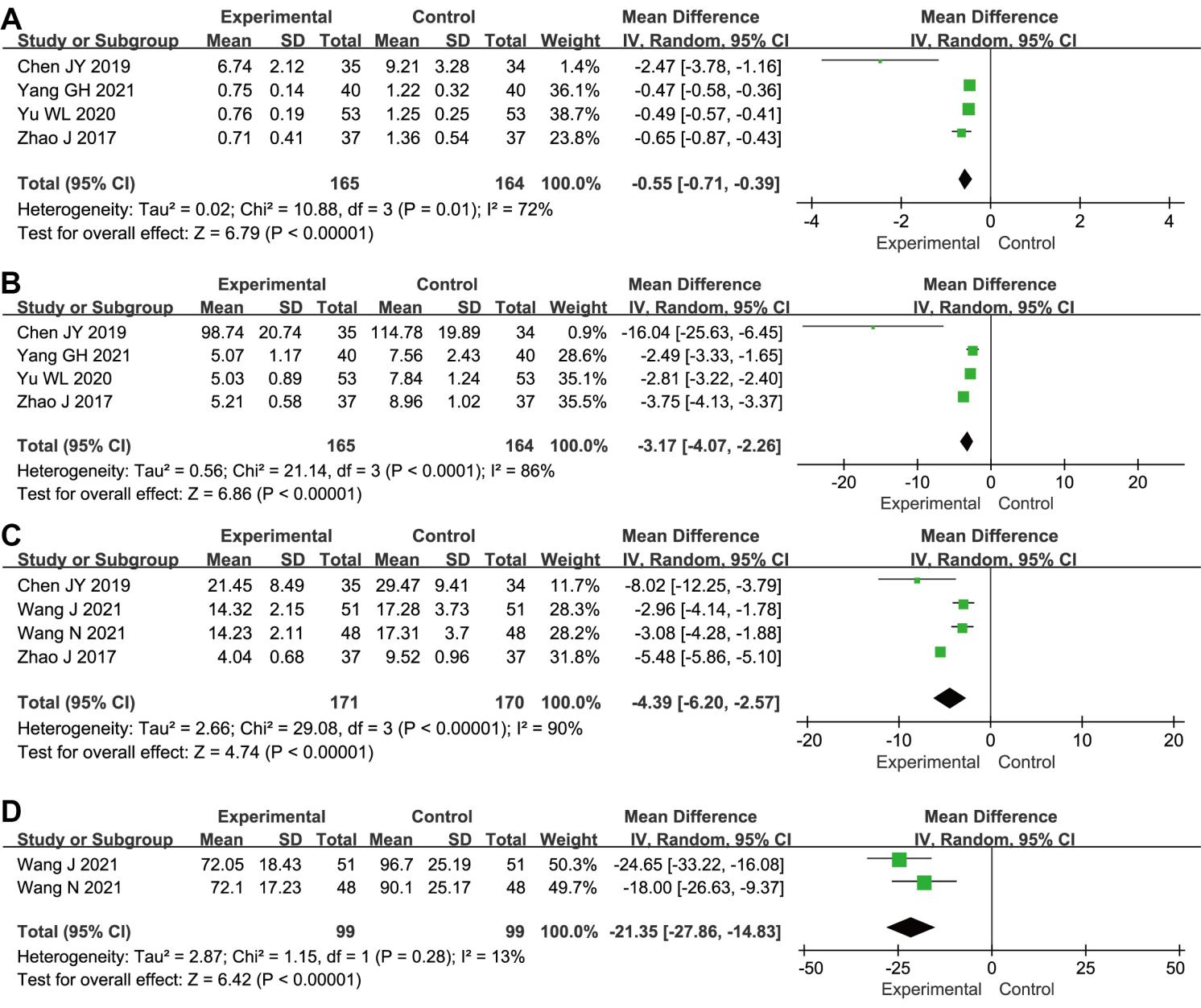
**

**Supplementary Figure S2.** Meta-analysis results for the intestinal barrier function of bifid triple viable preparation in acute ischemic stroke. (A) Endotoxin; (B) D-lactic acid; (C) Diamine oxidase; (D) Endothelin.

**
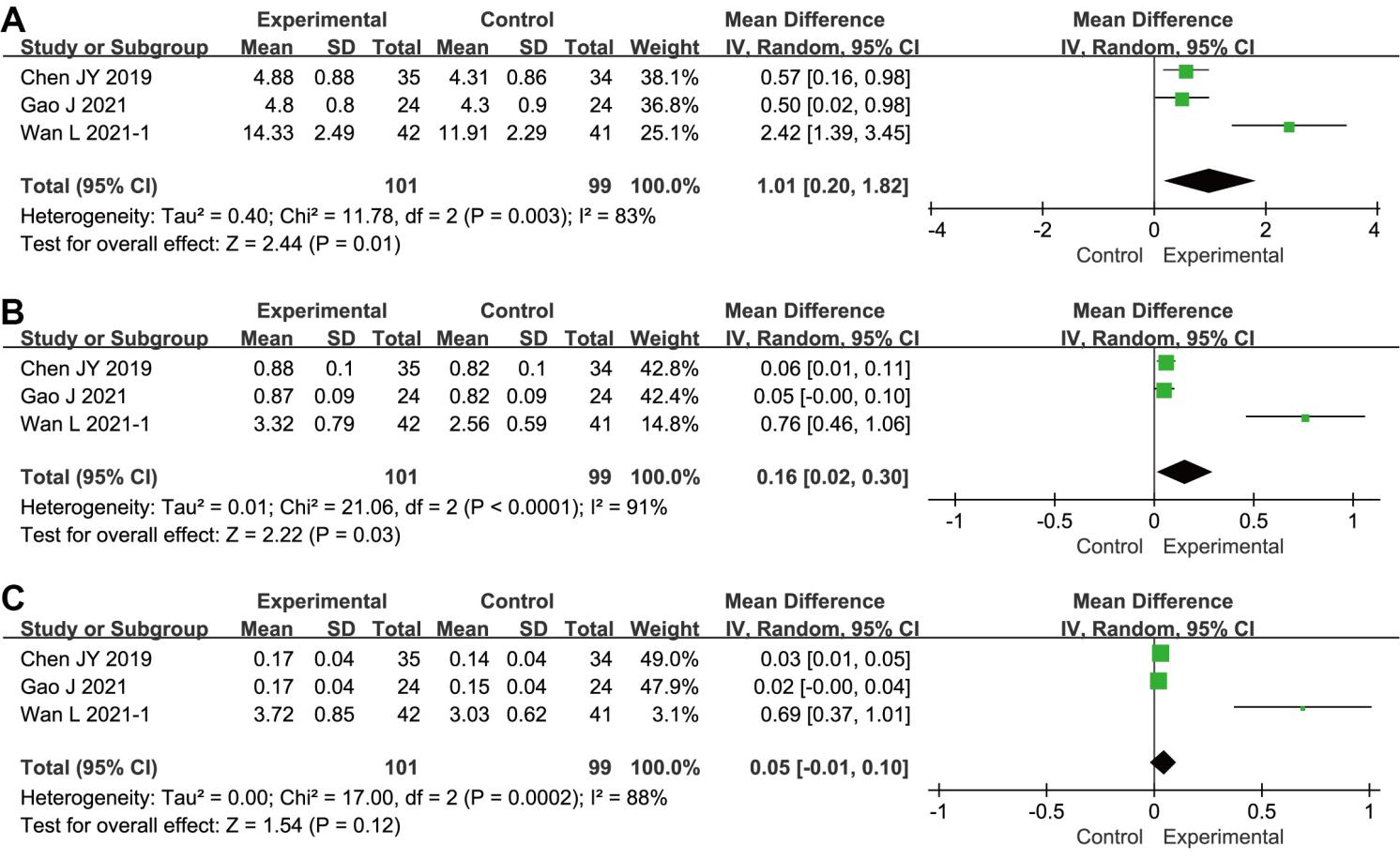
**

**Supplementary Figure S3.** Meta-analysis results for the immune function of bifid triple viable preparation in acute ischemic stroke. (A) IgG; (B) IgM; (C) IgA.
